# Book Notices

**Published:** 1867-01

**Authors:** 


### EDITORIAL.

### BOOK NOTICES.

*The Principles and Practice of Medicine.* By AUSTIN FLINT, M.D., Professor of the Principles and Practice of Medicine in the Bellevue Hospital Medical College, and in the Long Island College Hospital; etc., etc. Second edition, revised and enlarged. H. C. Lea, Philadelphia. 1867.

The success of the first edition of this excellent text-book has established its reputation. Anxious to improve its quality, the author tells us, in his preface to the second edition, that "the portion treating of pyæmia has been rewritten; three affections, omitted in the first edition, have been introduced, *viz.:*—pertussis, general cerebral paralysis, and polyuria; epidemic cholera has been considered at greater length; the thermometric phenomena of disease have received fuller consideration, and, in connection with many affections, there has been added new matter, much of which relates to special therapeutics."

For sale by W. B. Keen, 148 Lake St., Chicago.

---

*Notes on Epidemics: For the Use of the Public.* By F. E. ANSTIE, M.D., F.R..CP. Philadelphia: Lippincott & Co. 1866. pp. 95.

This is an expansion of an article by Dr. Anstie which originally appeared in the *British Quarterly Review.* It was designed to furnish "information which may assist the non-medical public to do their part in the work of preventing" those epidemic diseases which are the scourge of our cities. The little volume deserves a wide circulation.

For sale by S. C. Griggs & Co., 41 Lake Street.

---

*A Manual of Auscultation and Percussion.* By BARTH and ROGER. Translated from the sixth French edition. Philadelphia: Lindsay & Blakiston. 1866.

One of the most convenient little hand-books for students that we have seen. It will well repay careful study.

For sale by W. B. Keen & Co., 148 Lake Street, Chicago.

